# Study of the relationship between applied transmembrane pressure and antimicrobial activity of lysozyme

**DOI:** 10.1038/s41598-021-91564-x

**Published:** 2021-06-08

**Authors:** Simona M. Miron, Ariane de Espindola, Patrick Dutournié, Arnaud Ponche

**Affiliations:** 1 Institut de Science Des Matériaux de Mulhouse, Université de Haute-Alsace, CNRS IS2M UMR 7361, 3 bis rue A. Werner, 68098 Mulhouse Cedex, France; 2 Université de Strasbourg, Strasbourg, France

**Keywords:** Chemical engineering, Biomaterials - proteins, Chemical engineering

## Abstract

During the processing of biomolecules by ultrafiltration, the lysozyme enzyme undergoes conformational changes, which can affect its antibacterial activity. Operational conditions are considered to be one of the main parameters responsible for such changes, especially when using the same membrane and molecule. The present study demonstrates that, the same cut-off membrane (commercial data) can result in different properties of the protein after filtration, due to their different pore network. The filtration of lysozyme, regardless of the membrane, produces a decrease in the membrane hydraulic permeability (between 10 and 30%) and an increase in its selectivity in terms of observed rejection rate (30%). For the filtrated lysozyme, it appears that the HPLC retention time increases depending on the membrane used. The antibacterial activity of the filtrated samples is lower than the native protein and decreases with the increase of the applied pressure reaching 55–60% loss for 12 bar which has not been reported in the literature before. The observed results by SEC-HPLC and bacteriological tests, suggest that the conformation of the filtrated molecules are indeed modified. These results highlight the relationship between protein conformation or activity and the imposed shear stress.

## Introduction

In the last two decades, the application and processing of proteins in food, biological, pharmaceutical and chemistry industries have increased due to their use as food supplement (i.e. whey protein)^1, 2^, hormones, vaccines, antibiotics, antibodies and biopharmaceutical enzymes^3^, among others. Obtaining high quality proteins for delivery to the final consumers is a major concern of the industries and, for this reason, the processing of proteins is necessary. Some of applied unitary operations are purification, concentration, mixing, pumping, heating, and delivery. These procedure steps can induce protein denaturation and as a consequence the loss of biological activity^4, 5^. The common technique used to guarantee good quality of products is to add additives, preservatives, buffer, etc. However, these additional components can induce side effects.

Chromatography and membrane separations are commonly used for the purification of proteins during the processing^6^. The use of each is based upon the process and economic constrains. Using chromatography techniques, proteins can be purified considering their size, shape, total charge, hydrophobic groups at the surface and the binding capacity with the stationary phase. They are widely used due to their high degree of purity and recovery of protein^7^. However, chromatography is expensive, difficult to scale up or work with in batch operations^6^.

Membrane processes, on the other hand, are a greater alternative. Their advantages include low energy consumption, high selectivity, continuous possibility of separation, mild conditions of operation and the possibility to avoid any additional chemicals (additives). Another important feature is the possibility to adjust and vary the membrane properties in order to achieve the best fit for the process^8^. Besides the purification step, in order to produce a large quantity of purified proteins, the solution must be concentrated.

Ultrafiltration (UF) is a common choice for membrane process as it can be used concomitantly for protein concentration, buffer exchange, desalting, protein purification and viruses and bacteria clearance^9^. It is a pressure driven process for molecules with a molecular weight from 50 to 500 kg mol^−1^ and diameter of 1–100 nm^10^. Considering these values, it can retain solutes such as nanoparticles, colloids, and large molecules. Besides that, depending on the feeding solution, it is possible to choose different membrane materials varying from organic to inorganic ones. Inorganic membranes are more stable than organic ones at high temperature, pH conditions and limit the development of a biofilm^11^.

In UF processes, protein retention is very high but the efficiency of ultrafiltration can be affected by the characteristics of the membrane (material, pore size, surface charge, hydrophilicity), the protein solution (concentration, species, pH) and by the operating conditions (pressure, temperature, fluid velocity, etc.)^12^.

Even more, during the processing, the proteins are also subjected to changes in temperature, pH, pressures, to different organic solvents, to shearing, shaking etc. For example, during purification by membrane processes, the protein can be exposed to shear rates between 1000 and 10 000 s^−1^^13^. High shear can deform the three-dimensional structure of the protein and affect its inherent biological activity. Charm and Wong reported that by increasing the shear (shear rate 1155 s^−1^), the catalase enzyme suffers a decrease in activity up to 45%^14^. Bowen and Gan explained the loss of activity by the membrane-enzyme interaction resulting from the shear induced deformation of the enzyme structure during microfiltration^15^.

Lysozyme is a globular protein composed of 129 amino acids sequence and its molecular weight is approximately 14.6 kDa. It comes from hen egg white and is highly abundant in nature. It exhibits antibacterial action destroying the bacterial cell wall by polysaccharide hydrolysis. It is also used as an active ingredient in antivirus and anti-tumor drugs^16–18^.

Considering the possible influence of protein conformation on its biological activity, the aim of this work is to evaluate the behavior and antibacterial activity of lysozyme when ultrafiltration is used as a method for concentration. Different membranes and applied pressures were tested and antibacterial tests against *Micrococcus Lysodeikticus* were performed to understand the effect of processing conditions for protein applications.

## Materials and methods

### Materials

#### Chemical reagents

Vitamin B12 (98% purity) and Hen egg-white lysozyme (70.000 unit mg^−1^) were purchased from Alfa-Aesar and Sigma-Aldrich, respectively.

The evaluated concentrations were obtained by dissolving the desired amount into 4 L of demineralized water (18 MΩ). The concentration of each solution is presented in Table 1. All chemical reagents were used without any further purification.

**Table 1:** Basic information on the compounds used in the filtration experiments.

Compound	Molecular formula	Molar mass (g mol^−1^)	Stokes radius (nm)	Concentration (mol L^−1^)
Vitamin B12	${\text{C}}_{63}{{\text{H}}}_{88}{{\text{CoN}}}_{14}{{\text{O}}}_{14}{\text{P}}$	1355.38	0.7	9.22 × 10^–6^
Lysozyme	–	14,300	1.9	0.025 × 10^–3^

#### Membrane

The membranes chosen for the filtration experiments were mono-channel tubular, bilayer, asymmetric ultrafiltration membranes provided by TAMI Industries. These ceramic membranes are composed of γ-alumina support and an active layer of TiO_2_, with a cut-off of 1 kDa (manufacturer data) and isoelectric point at 6.0^19^. Considering that the real cut-off of the membranes can be different, experimental tests were conducted using several membranes. To illustrate this fact, filtration experiments were performed with three raw membranes (called M1, M2 and M3). The two former ones were also used after a cleaning process (the membrane was completely immersed in demineralized water and left in the oven at 105 °C for five consecutive days). These two membranes are called M1Reg and M2Reg. This protocol was used in order to recover initial hydraulic properties of the membrane, when it became saturated by protein adsorption.

### Experimental protocol

#### Filtration of protein solutions and membrane properties

Ultrafiltration tests were carried out in a laboratory pilot-plant provided by Techniques Industrielles Appliquées (TIA, Bolène, France) described in a previous work^20^. The pilot-plant operates in cross-flow mode with high flow velocity (700 L/h) in order to minimize the concentration polarization. Temperature was maintained constant (25 °C), while the pressure was varied from 4 to 12 bar. Samples from permeate (part of feed solution which pass through the membrane) and retentate (part of feed solution which is rejected by the membrane) were taken at each pressure for further analysis.

The performances of the membrane were assessed by filtration of demineralized water (hydraulic permeability) and filtration of vitamin B12 (selectivity performance) before and after the filtration of lysozyme (molecule of interest). Vitamin B12 had negligible effect on the membrane performances at the concentration used in the present study. Additional tests had confirmed that indeed there was no change in the membrane selectivity after successive filtrations of vitamin B12 (data not shown).

### Method of characterization

#### UV–VIS analysis

Retentate and permeate solutions were analyzed by UV spectroscopy (Lambda 35, Perkin Elmer Instrument) in order to determine the concentrations and the observed rejection rates. Each solution was analyzed at the wavelengths of 360 nm for Vitamin B12 and 280 nm for Lysozyme, respectively. Rejection rates were calculated using the following equation:

1 $$R_{obs} = \left( {1 - \left( {\frac{{A_{perm} }}{{A_{ret} }}} \right)} \right)*100$$

where *R*_*obs*_ is the observed rejection rate, *A*_*perm*_ is the absorbance of the permeate and *A*_*ret*_ is the absorbance of the retentate.

#### SEC-HPLC analysis

HPLC (High-Performance Liquid Chromatography) studies were performed with Agilent 1100 Series chain equipped with an UV detector and a quaternary pump. The column used for separation of proteins in the range of 400 000 to 4000 g mol^−1^ was a 9.4 × 250 mm Zorbax Bio Series GF-250 column (Agilent).

The buffer solutions for the mobile phase were prepared by dissolving 1 tablet of phosphate buffer saline (PBS from Sigma-Aldrich), 0.1% wt sodium dodecyl sulfate (SDS purchased from SIGMA-ALDRICH) and 0.005% wt sodium azide (NaN_3_) in 200 mL of demineralized water. The pH was 7.4 at 25 °C. The concentrations were 0.01 mol L^−1^ for phosphate buffer, 0.0027 mol L^−1^ for potassium chloride and 0.137 mol L^−1^ for sodium chloride.

100 µL of lysozyme solution was injected in the column and analyzed at the wavelength of 280 nm. The analysis was performed for 15 min, with a flow rate of 1.0 mL min^−1^ and a constant temperature of 25 °C.

##### Data treatment

Five imposed components (Gaussian shape, same full width at half maximum) was used to fit the chromatography peak profile. Each peak corresponded to a population of different hydrodynamic volume. The area of the peaks was considered positive and, then, curve fitting was performed by iterative least-squares calculation. Only the two major peaks (highest area of the 5 components) were considered for peak profile. The peaks were denominated as A and B, with A being the population with a lower hydrodynamic volume than B.

#### Validation of HPLC/UV–VIS calculation

Using Eq. 1 and according to the information from Huang et al.^21^, the rejection can also be calculated using the areas of the retentate and permeate obtained in HPLC experiments. In order to verify the results, the rejection rates obtained by the two methods were compared. The results from both techniques were in agreement (Supplementary data-Table S1).

#### Antibacterial activity of lysozyme analysis

Lysozyme antibacterial activity on the *Micrococcus Lysodeikticus* (ML) bacterial strain was studied using a microplate absorbance reader apparatus (MultiSkan FC from Thermo Fisher Scientific) and a 96 well microfiber sterile plate from Thermo Fisher.

*Micrococcus Lysodeikticus* (ML) lyophilized cells were purchased from SIGMA-ALDRICH. These tests were carried out according to Toro et al.^22^ and Lee et al.^23^. The assay started adding 20 µL of lysozyme solution in a well with 200 µL of bacteria culture (0.3 mg mL^−1^). The procedure was repeated twice with the same bacteria culture and the complete assay was performed two more times with different cultures to assess the reproducibility of the measurement. The solutions were stirred for 30 s before measurements and were incubated at 30 °C throughout the measurement.

Turbidity modification (ΔA_450nm_) was measured at 450 nm for 10 min with intervals of 15 s and the collected data was plotted as a function of time.

A pre-study was conducted for optimizing the method (investigation and results analysis) to study the protein antibacterial properties. Following the article of Prasad et al.^24^, the absorbance, the logarithm and the reciprocal of lysozyme absorbance against the bacteria substrate were plotted against time. The linearity for activity-time function was observed only in the plot of 1/Absorbance (1/A) as a function of time (data not shown). This indicated that the reaction between the lysozyme and the *Micrococcus Lysodeikticus* is a second order reaction.

The reaction rate was estimated from the slope of the 1/A_450nm_ versus time graph and the activity of the lysozyme samples was calculated using Eq. (2).

2 $${\text{A}}_{{\text{u}}} = \frac{{\frac{{{\text{Slope}}}}{{\left[ {{\text{LSZ}}} \right]}}}}{{\left[ {{\text{ML}}} \right]}}$$

where *A*_*u*_ is the activity of the lysozyme, Slope is the slope from 1/A_450nm_ vs. time graph, [LSZ] is the concentration of lysozyme in the well in mg mL^−1^, *[ML]* is the concentration of Micrococcus Lysodeikticus in the well in mg mL^−1^.

Normalization was done using the reference lysozyme sample (untreated lysozyme) value to give an index of activity Eq. (3).

3 $${\text{I}}_{{{\text{Au}}}} { } = { }\frac{{{\text{A}}_{{\text{u}}} }}{{{\text{A}}_{{{\text{u}}_{{{\text{reference}}}} }} }}{ }$$

where *I*_*Au*_ is the index of activity, *A*_*u*,_ *A*_*u native*_ are the calculated activities of the treated and the reference lysozyme, respectively.

##### Statistical analysis

In the current study, statistical test was performed with two pair t-test using OriginPro 2019 software. A confidence level of 95% was selected to estimate the significance and a difference of statistical significance was defined for *P* < 0.05.

## Results and discussion

### Filtration study

**Figure 1:**
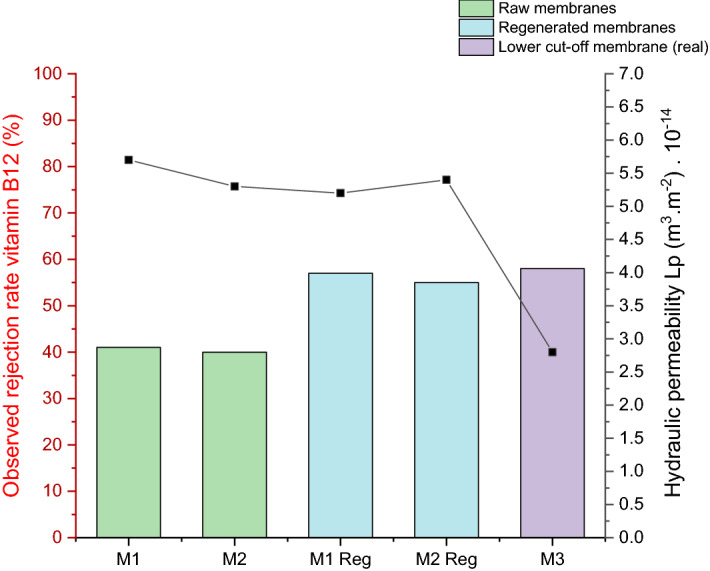
Initial membrane performances: hydraulic permeability Lp (point) and selectivity (columns) for the studied membranes (TAMI membranes, commercial cut-off 1 kDa).

Ultrafiltrations of lysozyme were carried out with raw membranes and regenerated ones following the protocol defined in §**2.2.2**. All of tested membranes have a commercial cut-off of 1 kDa. The initial properties, hydraulic (filtration of demineralized water) and selectivity performances (1st filtration of vitamin B12), of the chosen membranes for this study are presented in Fig. 1.

It can be observed that the membranes M1 and M2 have similar initial hydraulic permeability properties as M1Reg and M2Reg. However, the selectivity of M1Reg and M2Reg are slightly higher than for M1 and M2. This suggests that the membrane cleaning used in this study managed to restore the hydraulic performances of the membrane, but not the selectivity. The results show that the membranes have an apparent different pore size distribution (different rejection rates of vitamin B12). In a previous study^4^, it was observed that during the ultrafiltration of lysozyme, there was a decrease in the hydraulic performances of the membrane, while there was an increase in the selectivity. This behavior was related to a phenomenon of protein adsorption. Thus, considering all above, it can be presumed that a part of lysozyme was irreversibly adsorbed during filtration. While the membrane was cleaned, the irreversible adsorbed lysozyme was not removed, which might explain the increase in the rejection rate of vitamin B12.

M3 is a raw membrane with the same commercial cut-off as the other membranes used in this study (M1 and M2). However, during filtration tests, it was observed that it possesses different initial membrane properties. It had almost 50% less initial hydraulic permeability and it registered higher initial selectivity by 16 percentage points. Given its initial properties, this membrane had a lower real cut-off than the ones.

The different properties of the membranes tested were also highlighted by the selectivity towards the filtration of lysozyme. With M1 or M2, the rejection rate of the first lysozyme solution filtrated was around 80%. With M1Reg or M2Reg, the rejection rate of lysozyme was 90–95%. With M3, the rejection rate was 98%. Regardless of the membrane used, after filtration of lysozyme there was still an increase in selectivity and a decrease in permeability, suggesting adsorption of lysozyme inside the pores.

### Chromatography study of filtrated solutions

**Figure 2:**
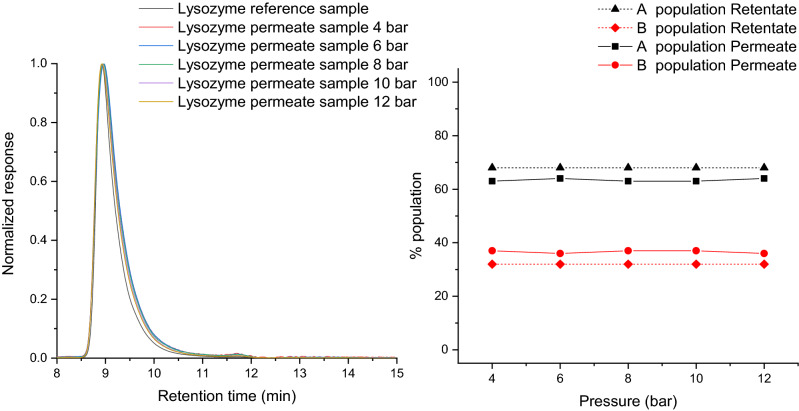
Normalized chromatograms of permeate samples (left) and population distribution (right) for M1 membrane.

The normalized chromatograms of permeate samples obtained when using raw membrane (M1 or M2) are presented in Fig. 2.

The results show that, when lysozyme was filtrated with a membrane M1 there was relatively no change in the hydrodynamic volume of the proteins after filtration regardless of the pressure used (Fig. 2 left). The same results were obtained with membrane M2. The population densities of the permeate were the same as for the retentate and the reference samples (Fig. 2 right). The reference sample (non filtrated lysozyme solution) was characterized by two populations with different hydrodynamic radius. The two major populations used to fit the chromatogram peaks are termed A (65%) and B (35%). There can be three explanations for these SEC-HPLC results. First, the shear stress was not sufficient to provoke any noticeable changes in the lysozyme solutions after filtration. Second, lysozyme could be denatured while passing through the pores, but regained its initial conformation (reversible denaturation) in the permeate solution. The case of reversible denaturation of lysozyme due to shear was observed by Ashton et al*.*^25^ using a Taylor-Couette flow cell. Third, just a small number of proteins was denatured due to the passage into the little pores compared to the greater protein amount that passed through the big ones. In this case, the membrane pore size distribution had a majority of pores with diameters around 2 nm, with some pores surpassing even 4 nm^26^. Lysozyme has a Stokes radius of 2 nm, thus, it passed through the larger pores without changing its three-dimensional folding structure. The shear forces in the larger pores were not sufficiently high to produce any irreversible changes in the lysozyme molecule after filtration.

**Figure 3:**
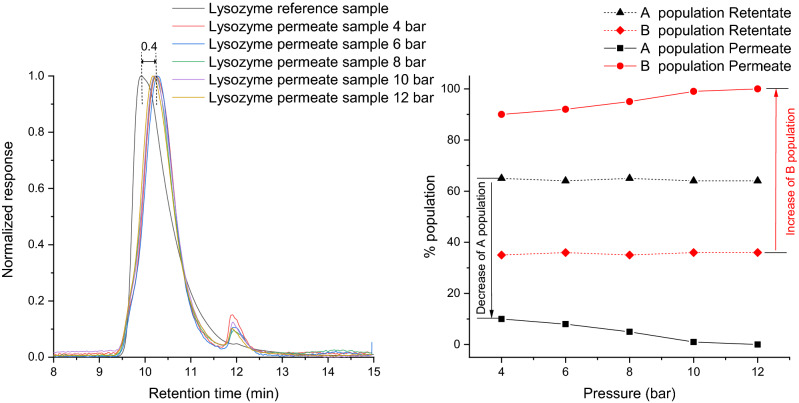
Normalized chromatograms of permeate samples (left) and population distribution (right) for M1Reg and/or M2Reg membranes.

The permeate solution filtrated with membranes M1Reg and M2Reg exhibited a delay in the retention time of 0.4 min comparing to the reference (Fig. 3 left). This indicated that there was a decrease in the apparent hydrodynamic volume of the lysozyme after filtration, which could be related to a breakage of intramolecular interactions and consequently, a change of hydration (denaturation) or a cleavage of disulfide bridges^27^. Nevertheless, the transmembrane pressure did not appear to cause any change in the hydrodynamic volume. On the other hand, the population densities changed for the filtrated samples. In Fig. 3 right it is observed that the A population in the permeate decreased from 65 to 10%. In the current case, the two membranes (M1Reg and M2Reg) have a different apparent pore size distribution with smaller pores when compared to M1 and M2. Thus, lysozyme was forced to pass through the pores by the applied pressure. Due to its dimensions and the pore size, the molecules were affected by a combination of interface interactions (e.g. electrostatic, dielectric, etc.) and shear forces.

Lysozyme filtrated with the lower real cut-off membrane (M3), also showed the same change in the hydrodynamic volume (data not shown). The shift in the retention time was towards higher values and was constant regardless of the applied pressure. The samples showed mainly a B population (99%) independently of the applied pressure. In this case, the forces acting upon the molecules managed to force lysozyme into a complete change of population. The behavior of the permeate samples (shift in retention time to higher values, decrease of A population) could be considered similar with the behavior of the samples filtrated with M1Reg and M2Reg.

Thus, it can be concluded that it was the real cut-off / pore size, which contributed to the behavior of the lysozyme after filtration. Shear and interactions between proteins and surface also modified the conformation of the filtrated lysozyme.

Marieke van Audenhaegue et al. showed that the cut-off of the membrane was mainly responsible for the modifications of the protein after filtration (ultrafiltration of α-lactalbumin 14.2 kDa^28^. In the study of Portugal et al.^29^, it was concluded that it was the ratio between the protein size and the membrane pore size which influenced the behavior of the molecule. If the pore size was bigger than the protein dimensions, shear stress influence was decreased, and any other physicochemical condition could be considered as responsible for the modifications observed in the molecule. If the pore size was much smaller than the protein, then the structural modifications occurring to the molecule could be attributed to shear stress.

### Antibacterial activity of filtrated solutions

**Figure 4:**
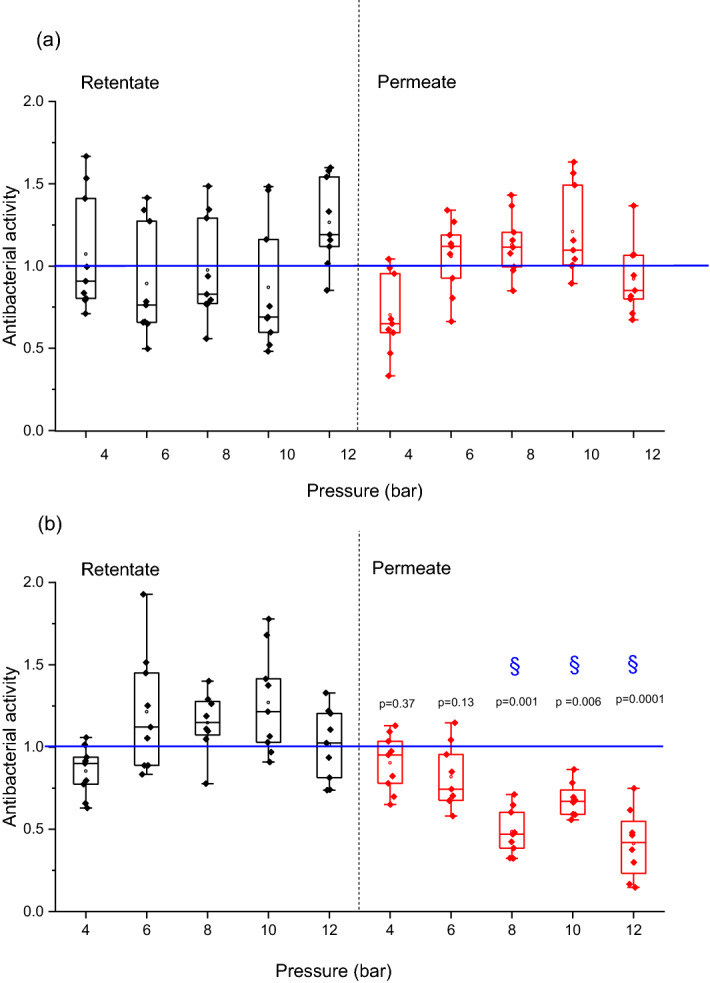
Lysozyme antibacterial activity for retentate (black) and permeate (red) solutions for (**a**) raw (M1 and M2) and (**b**) M1Reg and M2Reg; § represents the significant difference for *P* < 0.05 with respect to lysozyme reference (untreated lysozyme), blue line represents the lysozyme reference.

For the membranes M1 and M2, the permeate and retentate samples (Fig. 4a) showed (statistically) the same activity against Micrococcus Lysodeikticus comparing to the reference sample. For the membranes M1Reg and M2Reg (Fig. 4b), the retentate showed no modification of antibacterial activity due to recirculation. The antibacterial activity of permeate samples appeared to decrease with the increase of pressure. The change in activity was statistically significant (*P* < 0.01) at a pressure higher than 8 bar and it decreased by 60% for 12 bar.

**Figure 5:**
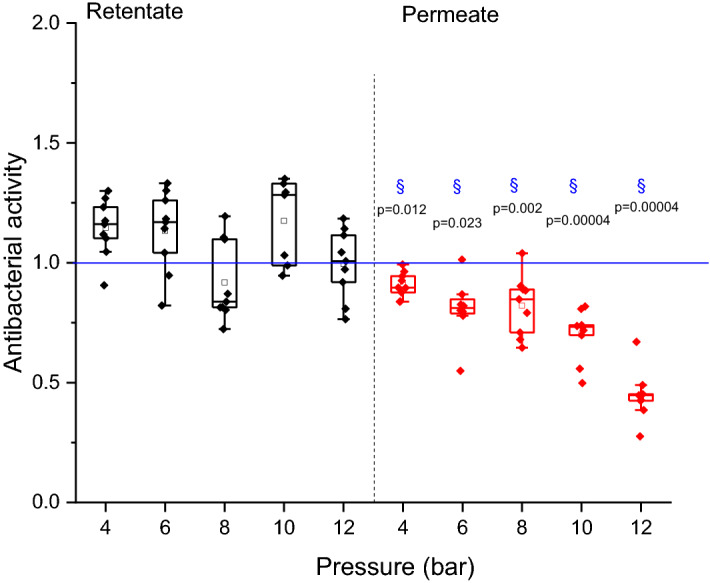
Lysozyme antibacterial activity for retentate (black) and permeate (red) solutions for lower cut-off membrane M3; § represents the significant difference for *P* < 0.05 with respect to lysozyme reference (untreated lysozyme), blue line represents the lysozyme reference.

Results for M3 (Fig. 5) were in agreement with M1Reg and M2Reg observations. There was also a statistical decrease in antibacterial activity with pressure increase. At 12 bar pressure, lysozyme lost 55% of its activity.

In the literature there is not any study showing a correlation between applied pressure and a decrease of protein activity after filtration process. Lesnierowski et al. had studied the effect of applied pressure, temperature, pH and ultrafiltration time on antibacterial activity of lysozyme^30^. They did not observe a change due to the pressure increase, but only due to a change of pH.

The changes in hydrodynamic properties and antibacterial activity are related to the operating conditions and to pore size distribution of the membrane used. The results suggest that the filtration with a low cut-off membrane caused sufficiently high shear stress in the pores to change protein conformational structures and to modify its inherent bactericidal activity.

## Conclusion

A relationship between the hydrodynamic parameters, protein conformation and cell lysis activity of lysozyme was investigated in this work by ultrafiltration experiments performed at different pressures, SEC-HPLC analysis and antibacterial tests.

With the lower “real” cut-off membranes, the filtrated protein underwent modifications related to its hydrodynamic volume and showed a decrease in its antibacterial activity, indicating a significant denaturation. For the membrane with the higher cut-off, no significant difference was observed between filtrated and non-filtrated solutions (chromatography and antibacterial activity investigations). These results show the relationship between the protein properties and shear forces in the pore of the membrane. The antibacterial activity loss (up to 60% at 12 bar) was directly related with the increase of pressure (shear forces in the pore) that has never been reported in the literature. The membrane’s real cut-off should be taken into account when processing proteins especially in the pharmaceutical industry. Besides that, the choice of operating conditions for the optimization of the process should also be considered.

## Supplementary Information

Supplementary Information.
