# Supplementary material for: Study of the relationship between applied transmembrane pressure and antimicrobial activity of lysozyme
Source: Sci Rep. 2021 Jun 8;11:12086. doi: 10.1038/s41598-021-91564-x (PMC8187345; doi:10.1038/s41598-021-91564-x)
Supplement: Supplementary file 1 — Supplementary Information. [file 41598_2021_91564_MOESM1_ESM.docx]

Supplementary data

Table S1. Rejection rate (%) calculated by UV-VIS and by HPLC for lysozyme filtrated with a regenerated membrane (M1Reg / M2Reg)

| Pressure | R(%) by UV-VIS | R(%) by HPLC |
| --- | --- | --- |
| 4 | 95.7 | 95.3 |
| 6 | 94.0 | 94.3 |
| 8 | 91.2 | 91.9 |
| 10 | 91.7 | 91.6 |
| 12 | 90.7 | 91.7 |
